# Design of a Photo-Assisted Electrochemical Potentiostat for Preconcentration-Free Detection of Heavy Metal Copper Ions

**DOI:** 10.3390/s26144411

**Published:** 2026-07-11

**Authors:** Yuhan Hao, Haiyang Huang, Cong Zhao, Yihao Geng, Yingmao Luo, Chun Zhao, Hui Suo

**Affiliations:** 1 State Key Laboratory of Integrated Optoelectronics, JLU College of Electronic Science and Engineering, Jilin University, Changchun 130012, China; 2 Jilin Province Product Quality Supervision and Inspection Institute, No. 2699, Yiju Road, Changchun 130103, China

**Keywords:** photo-assisted electrochemistry, potentiostat, heavy metal detection, preconcentration-free, analog front-end

## Abstract

Rapid on-site detection of heavy metal ions is important for food and water safety, while traditional electrochemical stripping voltammetry is limited by its time-consuming preconcentration step. Herein, an integrated photo-assisted electrochemical potentiostat, PAEcStat, was developed for the rapid preconcentration-free detection of heavy metal ions. The system integrates a 20 W LED driver with closed-loop light-intensity regulation and a low-noise electrochemical analog front-end for differential pulse voltammetry. The illumination intensity can be regulated from 1000 to 19,000 lux at a distance of 10 cm with an error within ±100 lux. The analog front-end provides a current detection range of 10 nA to 4 mA, with excellent linearity (R^2^ = 0.99991) and a DPV current deviation of only 0.063% in simulated tests. For sensing validation, a photoactive Co_3_O_4_/WO_x_/FTO electrode was fabricated for Cu^2+^ detection under blue-light illumination, achieving a calibration equation of I_pa_ = 50.28C + 8.96 with R^2^ = 0.984. Compared with a commercial CHI660E workstation, PAEcStat showed an average relative deviation of 1.92% over 0.1–1.0 mg/L Cu^2+^. These results demonstrate that PAEcStat is a reliable and integrated platform for rapid photo-assisted electrochemical detection in on-site applications.

## 1. Introduction

Driven by rapid global industrialization and urbanization, heavy metal pollution has emerged as one of the most severe environmental and public health challenges facing human society today [1]. The improper practices in mineral extraction, metal smelting, chemical manufacturing, and the application of agricultural fertilizers have led to the continuous release and accumulation of heavy metal ions—such as lead, cadmium, copper, and mercury—in natural water bodies and soils [2]. These heavy metals are essentially non-biodegradable, meaning that they can bioaccumulate through the food chain and ultimately enter the human body [3]. Long-term exposure to these elements causes irreversible damage to the nervous system, kidneys, and skeletal system [4]. Even copper ions, which are essential trace elements for the human body, can induce severe cellular oxidative stress upon excessive long-term accumulation, subsequently leading to hepatic and renal dysfunction, and even neurodegenerative diseases [5,6]. Consequently, the World Health Organization (WHO) and various national regulatory agencies have established highly stringent standard limits for heavy metals in water bodies and food [7].

Standardized methods for determining total heavy metal concentrations, such as inductively coupled plasma-mass spectrometry (ICP-MS) [8] and atomic absorption spectroscopy (AAS) [9], heavily rely on large-scale laboratory instruments. These methods typically suffer from bulky equipment and exorbitant costs, and their detection cycles usually exceed several hours, rendering them unsuitable for rapid, on-site detection. In contrast, electrochemical methods have been widely applied for the on-site detection of heavy metal ions due to their unique advantages: simple instrumentation, low power consumption, ease of miniaturization, and the capability to achieve trace-level sensitivity [10,11,12], achieving detection performances approaching those of spectroscopic methods in drinking water, natural water, and food digestion solutions [13].

The most commonly used electrochemical method for detecting heavy metal ions is stripping voltammetry. However, due to its reliance on a preconcentration step, a single test usually requires minutes or even longer for the deposition time, which significantly restricts detection throughput and real-time monitoring capabilities. To overcome the time bottlenecks and operational complexities introduced by the preconcentration step in traditional stripping voltammetry, researchers have dedicated efforts to developing preconcentration-free detection schemes [14,15]. Transition metal oxides possess unique valence cycling characteristics, allowing them to participate directly in the redox cycling of target ions. By leveraging their own valence state transitions, they can directly provide electrons to heavy metal ions for spontaneous reduction, eliminating the need for an externally applied negative potential from the instrument for electroreduction. This mechanism enables the preconcentration-free detection of heavy metal ions [16,17,18]. While this preconcentration-free electrochemical detection significantly shortens the assay time, its detection performance is slightly inferior to that of traditional stripping voltammetry with a preconcentration step, facing the challenge that the target signal is easily obscured by background noise. To address this, researchers have attempted to introduce photo-assisted technologies, where illumination at specific wavelengths excites semiconductor photoelectrodes to generate photogenerated electron–hole pairs [19,20]. These highly active carriers induce efficient direct charge transfer at the electrode surface, directly driving the reduction reaction of heavy metal ions [21]. Simultaneously, the strong reducing ability of photogenerated carriers can significantly amplify the Faradaic peak current [22], thereby drastically improving the signal-to-noise ratio (SNR) without increasing the background current. This ensures that even when the lengthy physical electrodeposition preconcentration step is eliminated, sufficiently resolvable stripping signals can still be obtained under low-concentration conditions.

Although photo-assisted electrochemical methods exhibit outstanding potential for signal enhancement and background suppression, the vast majority of current research remains at the laboratory validation stage, relying heavily on commercial benchtop electrochemical workstations paired with independently configured external light sources [23]. This combinational setup is not only bulky and complex to wire, but also suffers from exceptionally high power consumption and a strict dependence on a stable indoor environment. This severely limits the universal applicability and detection efficacy of photo-assisted electrochemical methods in diverse outdoor environments [24]. For photoelectrochemical (PEC) sensing, some researchers have attempted to integrate light sources into miniaturized instruments, demonstrating the feasibility of portable photoelectric detection [25]. Cao et al. developed a miniaturized PEC measurement system using a discrete Fourier transform (DFT) demodulation algorithm, combined with a molecularly imprinted polymer (MIP) sensor for the detection of macromolecules such as microcystin-LR [26]. Zheng et al. designed a novel portable PEC analyzer powered by a power bank, integrating micro light-emitting diode (LED) excitation with a Universal Serial Bus (USB) electrochemical workstation, and utilized it alongside an aptasensor for detecting *E. coli* O157:H7 [27]. Alexander Scott et al. introduced a portable PEC reader device called PECsense, which can be operated remotely via smartphone Bluetooth Low Energy (BLE) [28]. It synchronously controls LED light source excitation and executes chronoamperometry for electrochemical readouts. Paired with surface-modified titanium dioxide photoelectrodes, this system was successfully applied to the biosensing detection of DNA hybridization. Anh Hao Huynh Vo et al. developed P-EcStat, a novel multi-functional electrochemical and photoelectrochemical (EC-PEC) dual-mode sensing measurement system that connects to a smartphone via BLE and integrates a multi-wavelength monochromatic LED excitation array [29]. Combined with hydrothermally synthesized copper oxide (CuO) nanorods as photoactive materials, the system’s performance was validated through the redox detection of a potassium ferricyanide/ferrocyanide solution. However, most existing portable potentiostats are designed solely for conventional electrochemical techniques and lack coordinated photoelectrochemical control. Furthermore, currently reported portable PEC instruments rely on low-power, non-replaceable light sources, making them inadequate for photoactive materials demanding high illumination intensities.

To fill this gap, this paper presents PAEcStat, a portable potentiostat specifically customized for photo-assisted electrochemical analysis. It integrates a high-power LED driver circuit explicitly designed for the preconcentration-free, on-site detection of heavy metal copper ions. Its hardware architecture combines a low-noise electrochemical analog front-end (AFE) optimized for differential pulse voltammetry (DPV) with a closed-loop light intensity regulation system driven by an incremental proportional-integral-derivative (PID) algorithm, achieving precise light irradiation directly at the target sensing location. Wi-Fi communication facilitates convenient wireless parameter configuration and real-time concentration display. Using a photoactive Co_3_O_4_/WO_x_/FTO working electrode, the system successfully accomplished the direct, preconcentration-free detection of copper ions in real water samples. Calibration against a commercial benchtop workstation under identical blue-light excitation validated its measurement accuracy. Ultimately, this work provides a unique engineering architecture that bridges the gap between laboratory-based modular PEC setups and practical, field-deployable instruments.

## 2. Materials and Methods

### 2.1. Background of Photo-Assisted Electrochemical Sensing

**Figure 1:**
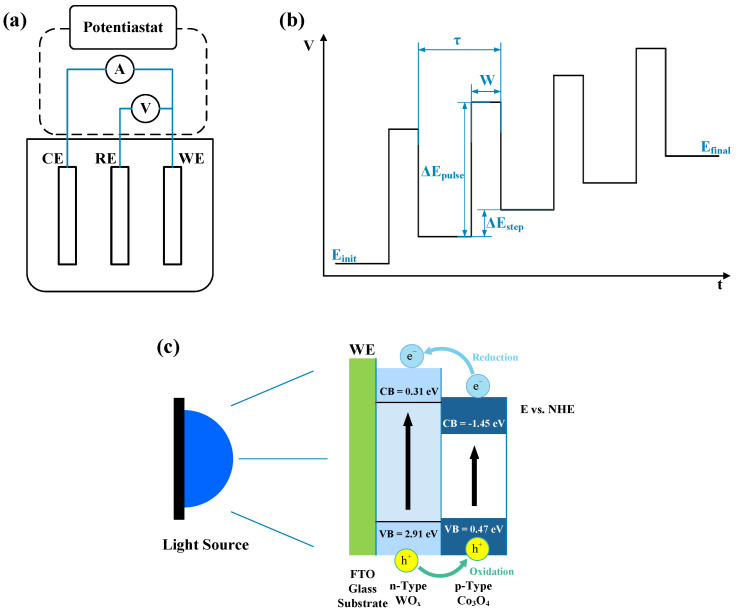
(**a**) Working principle of the three-electrode system. (**b**) Excitation signal waveform of DPV. (**c**) Principle of photo-assisted electrochemistry.

Conventional electrochemical sensing methods are typically constructed based on a three-electrode system [30], which primarily comprises a working electrode (WE), a reference electrode (RE), and a counter electrode (CE). As depicted in Figure 1a, the WE serves as the sensing electrode, acting as the primary interface where the redox reactions of target ions occur. The RE is utilized to provide a reference potential, ensuring that the voltage applied between the WE and RE remains stable and controllable. Meanwhile, the CE and WE form a current loop for charge transfer. By analyzing how the response current within this loop varies with the applied potential or time, the concentration of target ions in the solution can be quantitatively determined.

Depending on the excitation signal waveforms applied by the AFE, common electrochemical analysis methods include cyclic voltammetry (CV), linear sweep voltammetry (LSV), chronoamperometry (i-t), and differential pulse voltammetry (DPV) [31]. Among these, DPV is frequently employed for heavy metal ion detection. Figure 1b illustrates the excitation signal waveform of DPV, which is typically formed by superimposing a periodic pulse wave onto a base staircase wave. This waveform is defined by six parameters: initial potential, final potential, potential step, pulse amplitude, pulse period, and pulse width. Current sampling is conducted at the end of the low-level and high-level states of each pulse, respectively. The difference between these two sampled values is then utilized as the effective current signal to plot the voltammetric characteristic curve. This differential sampling mechanism can substantially suppress interference from the background current, thereby significantly enhancing the signal-to-noise ratio (SNR) and detection sensitivity for weak signals [32].

Photo-assisted electrochemical methods represent an advancement over traditional electrochemical detection. In preconcentration-free detection systems, employing photoactive metal oxides as the working electrode (WE) can compensate for the sensing performance of the sensitive electrode [33]. Specifically, WO_x_ possesses excellent visible-light response and stable semiconductor characteristics, providing an effective platform for the generation and transport of photogenerated charge carriers. Meanwhile, Co_3_O_4_ exhibits a reversible Co^2+^/Co^3+^ redox couple, which facilitates interfacial charge transfer and promotes the electrochemical reaction of target metal ions. Furthermore, the heterojunction formed between Co_3_O_4_ and WO_x_ effectively enhances the separation efficiency of photogenerated electron–hole pairs while suppressing charge recombination, thereby improving the photo-assisted electrochemical sensing performance. As depicted in Figure 1c, under illumination, electrons in the valence band of the metal oxide transition to the conduction band, generating an abundance of photogenerated electron–hole pairs [34]. This not only improves the surface charge transfer efficiency of the electrode material, but also enhances its electrocatalytic reduction/oxidation activity toward target analytes, thereby substantially elevating sensing performance. Benefiting from this photo-assisted enhancement effect, this method can bypass the time-consuming preconcentration step typically required in traditional electrochemical detection. Under illumination, a DPV signal scanning from a negative potential to a positive potential is directly applied to the WE. During the positive potential scan, target ions adsorbed on the electrode surface generate characteristic stripping current peaks at their specific oxidation potentials. The characteristic potential at which this peak appears can be utilized to qualitatively identify the specific analyte. Simultaneously, within a certain range, the amplitude of the stripping current peak exhibits a strong linear relationship with the concentration of the target ions in the solution. Based on this relationship, the precise quantification of the target ion concentration can be achieved by fitting a standard working curve.

### 2.2. System Design

**Figure 2:**
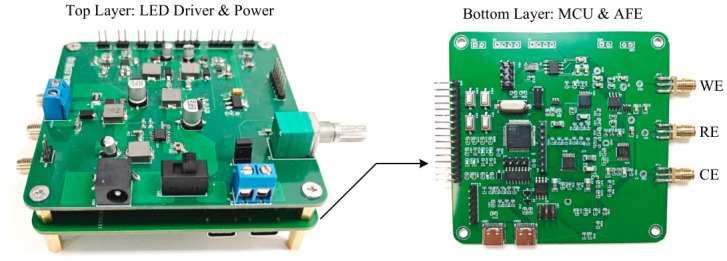
The designed PAEcStat system primarily comprises two parts: the electrochemical AFE and the light source driver circuit.

The system design of the PAEcStat primarily comprises two components: an electrochemical AFE for scanning voltage application and current signal measurement, and a high-power LED constant-current driver circuit for light intensity regulation. Figure 2 illustrates the conceptual system of the designed PAEcStat, which is composed of two custom-designed printed circuit boards (PCBs).

#### 2.2.1. Electrochemical Analog Front-End

**Figure 3:**
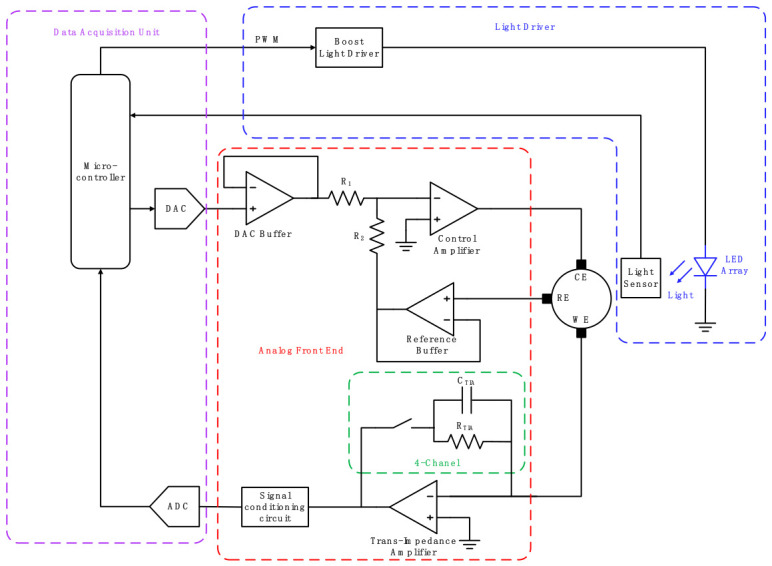
Schematic diagram of the overall structure of the PAEcStat, which primarily consists of a light source module and an electrochemical AFE.

To achieve high-sensitivity and low-noise electrochemical detection of trace heavy metal ions via DPV, a high-precision electrochemical AFE was designed and implemented. Figure 3 presents the detailed schematic block diagram of the developed potentiostat and signal chain. This architecture primarily consists of three core functional modules: a potentiostatic control loop, a programmable gain transimpedance amplifier (TIA), and a signal conditioning circuit.

The primary objective of the potentiostatic control loop is to precisely regulate the potential difference between the RE and the WE, ensuring that it strictly tracks the excitation signal *V_DAC_* generated by the digital-to-analog converter (DAC). The control loop consists of a cascaded DAC buffer, a control amplifier (CA), and a reference voltage buffer. The DAC buffer utilizes a low-noise operational amplifier, OPAx392 (Texas Instruments, Dallas, TX, USA), configured as a voltage follower to buffer the input *V_DAC_*. The OPAx392 provides an extremely high input impedance and an ultra-low output impedance, effectively isolating the DAC voltage source and eliminating polarization errors caused by downstream loading effects. A zero-drift precision operational amplifier, OPAx189 (Texas Instruments, Dallas, TX, USA), is employed as the CA. Owing to its wide supply voltage range of 4.5 V to 36 V and an output current of ±65 mA, it can provide a broader current detection range for the subsequent TIA. Furthermore, benefiting from its chopper-stabilized architecture, the 1/f flicker noise is virtually eliminated at low frequencies, and the offset voltage is as low as 0.4 μV, thereby guaranteeing outstanding DC precision and long-term stability during the electrochemical potential scanning process. The output of the CA is connected to the CE to dynamically maintain the electrochemical cell current, ensuring that the feedback potential at the RE precisely converges to the target value via the inverting summing topology structured by *R*_1_ and *R*_2_. A high-impedance voltage follower based on the OPAx392 is adopted as the reference buffer. This can effectively prevent leakage currents from causing polarization at the RE, ensuring the lossless capture of the reference potential and its feedback into the potentiostatic control loop. At this point, the voltage difference *V_WR_* between the WE and RE can be determined by Equations (1) and (2):

(1) $$\frac{V_{DAC}}{R_{1}}+\frac{V_{RE}}{R_{2}}=0$$

(2) $$V_{WR}=V_{WE}-V_{RE}$$

A programmable gain TIA is designed to broaden the measurement range for weak currents. The TIA utilizes the OPAx392, which features a femtoampere-level ultra-low input bias current, to minimize errors introduced by the amplifier’s input stage. The inverting input terminal of the operational amplifier is connected to the WE to form a weak current detection unit, maintaining a virtual ground state to ensure that the Faradaic current generated by the redox reactions flows entirely into the I–V converter. To accommodate the wide dynamic range of stripping currents associated with different analyte concentrations, a low on-resistance analog switch, ADG1604 (Analog Devices, Inc., Norwood, MA, USA), is integrated to dynamically switch the gain feedback resistors via programming for range selection. The feedback resistor *R_TIA_* is divided into four ranges from 470 Ω to 470 kΩ, and the electrochemical cell current can be determined by Equation (3):

(3) $$I_{cell}=\frac{V_{ADC}}{R_{TIA}}$$

A fourth-order multiple feedback (MFB) low-pass filter is designed as the signal conditioning circuit. Since the analytical voltammetric peaks of heavy metal ions are inherently low-frequency signals, the raw TIA output *V_TIA_* inevitably contains high-frequency environmental interference, switching noise from the analog switch, and broadband thermal noise from the pre-stages. To achieve superior out-of-band rejection and signal purification, a fourth-order Butterworth-Bessel active low-pass filter is implemented using the OPAx392 by cascading two second-order MFB topologies. The MFB structure was selected due to its low sensitivity to component tolerances and robust stopband attenuation characteristics. Through the configuration of the RC network, the cutoff frequency f_c_ is tuned to 140 Hz. This filter can effectively suppress high-frequency electronic noise while preserving the intact morphology of the low-frequency voltammetric waveforms, acting as an optimized anti-aliasing filter to provide a pure analog signal *V_ADC_* to the subsequent high-resolution analog-to-digital converter (ADC).

For excitation waveform generation, a 16-bit high-precision DAC, the LTC2642 (Analog Devices, Inc., Norwood, MA, USA), is employed. It functions in conjunction with a 2.048 V stable reference source and a zero-drift operational amplifier, OPAx189, to accomplish level shifting, thereby providing a precise, low-noise bipolar voltage excitation *V_DAC_* with an output voltage range of −2.048 V to +2.048 V. In the data acquisition path, a 24-bit high-resolution Δ-Σ ADC, the ADS1246 (Texas Instruments, Dallas, TX, USA), is utilized. It similarly uses a 2.048 V reference voltage source to accurately offset the current acquisition range to between −2.048 V and +2.048 V for sampling the filtered electrochemical response signal *V_ADC_*. Both the DAC and ADC communicate with the microcontroller unit (MCU) via a high-speed Serial Peripheral Interface (SPI) bus. The main control unit adopts an STM32 microcontroller (STMicroelectronics, Geneva, Switzerland), which is responsible for excitation waveform output, precise timing synchronization between current sampling points, and data processing.

#### 2.2.2. Light Source Driver

The illumination system of the PAEcStat adopts a closed-loop feedback control architecture, primarily comprising the MCU, the LED driver circuit, and a light intensity detection loop. The principle of this control system is illustrated in Figure 3. After receiving and parsing commands, the MCU utilizes its internal advanced timer to generate a high-frequency pulse-width modulation (PWM) signal. The LED driver employs a step-up (Boost) constant-current driver chip, SY7203 (Silergy, Hangzhou, China). This chip can boost the front-stage input voltage to the 12 V operating voltage required by the LED, and controls the LED drive current via the input PWM duty cycle, with a theoretical maximum drive current reaching 1.67 A. The light source consists of a series-parallel combination of 120 blue LEDs. The combined LED (Shenzhen Ruicheng Optoelectronics Technology Co., Ltd., Shenzhen, China) operates at a voltage of 12 V, with a central wavelength ranging from 460 nm to 470 nm, and a viewing angle of 130°. The light intensity feedback loop is constructed based on a BH1750 (ROHM Semiconductor, Kyoto, Japan) digital light sensor. The sensor directly converts the real-time captured actual light irradiance into a digital signal and feeds it back to the MCU via the Inter-Integrated Circuit (I2C) bus.

Regarding the closed-loop control algorithm, this system employs an incremental PID control algorithm to adjust the output PWM duty cycle in real-time, achieving better control over the illuminance in the target area. By calculating the increment of the control variable in the current sampling period, the incremental PID algorithm circumvents the risk of integral windup inherent in traditional positional algorithms. Simultaneously, because the duty cycle is only adjusted within a limited step size each time, the software and hardware safety of the control system is greatly enhanced. This effectively eliminates the hidden danger of chip damage caused by drive voltage overshoot resulting from abrupt algorithmic variations or signal disturbances.

#### 2.2.3. Data Processing Algorithm

To eliminate the interference of background charging current and high-frequency noise, the system embeds digital signal processing algorithms within the MCU to achieve the precise extraction of characteristic stripping peaks. Current values are sampled at the end of the high-level and low-level pulses of the DPV waveform, respectively, and their difference is calculated to obtain the net current value as the effective signal. Prior to testing, the system executes a DPV scan on a blank buffer solution in advance and saves the measured background baseline data. During actual detection, the corresponding baseline data are subtracted from the acquired voltammetric data, thereby purifying the stripping signal of the target ions. Subsequently, a Savitzky–Golay (S–G) filter based on a cubic polynomial is employed to smooth the net current curve [35]. With a window size of 11, the filter effectively eliminates high-frequency spikes while maximally preserving the true height and morphology of the stripping peaks.

After obtaining the smoothed curve, the system executes a differential peak-finding algorithm to extract the peak current. The algorithm first calculates the first derivative of the smoothed signal to locate local extrema by finding the zero-crossing points transitioning from positive to negative. Next, combined with the criterion that the second derivative is less than zero, the extremum is confirmed as the peak of the current, thereby eliminating the interference of valleys and inflection points. To thoroughly circumvent false peaks caused by residual fluctuations, a threshold constraint is introduced. An extremum is judged as a valid peak only when the net current at the extreme point exceeds a set limit, and the voltage span extending to the valleys on both its left and right sides conforms to the characteristics of a typical heavy metal stripping peak. Finally, the system extracts the validated effective peak current and substitutes it into a pre-established “peak current–ion concentration” standard working curve for linear fitting calculation, automatically deriving and outputting the precise concentration of the target heavy metal ions in the water sample.

### 2.3. Chemical Reagent

All chemical reagents used in this study were of analytical grade and used as received without further purification. FTO conductive glass was purchased from Luoyang Guluo Glass Co., Ltd. (Luoyang, China). Toluene and acetone were obtained from Xilong Scientific Co., Ltd. (Shantou, China). Ethanol and hydrogen peroxide (H_2_O_2_, 30%) were provided by Sinopharm Chemical Reagent Co., Ltd. (Shanghai, China). Tungstic acid (H_2_WO_4_) was acquired from Shanghai Macklin Biochemical Co., Ltd. (Shanghai, China). Ammonium chloride (NH_4_Cl) and glacial acetic acid were purchased from Beijing Chemical Factory Co., Ltd. (Beijing, China). Cobalt nitrate hexahydrate (Co(NO_3_)_2_·6H_2_O) was purchased from Aladdin Reagent (Shanghai, China) Co., Ltd. (Shanghai, China). Ethylene glycol was obtained from Tianjin Tiantai Chemical Co., Ltd. (Tianjin, China). All aqueous solutions were prepared using deionized water.

### 2.4. Electrode Preparation

Co_3_O_4_ and WO_x_ materials exhibiting photoactive responses were selected to fabricate the sensitive electrode, and the preparation method referred to the in situ growth process [36]. First, FTO conductive glass with dimensions of 1 cm × 2 cm was sequentially immersed in toluene, acetone, ethanol, and deionized water, undergoing sequential ultrasonic cleaning for 10 min each to obtain a pristine deposition interface. Then, 1.8 g of tungstic acid (H_2_WO_4_) was dissolved in 35 mL of deionized water and 5 mL of H_2_O_2_ solution, and stirred for 4 h. Subsequently, the solution was heated to 95 °C and continuously stirred until homogeneous, after which it was diluted to 40 mL with deionized water. Next, 4 mL of the precursor solution was mixed with 1.5 g of NH_4_Cl and 16 mL of ethanol and transferred into an autoclave with a 100 mL Teflon liner. The FTO substrate was placed into the solution, and the autoclave was reacted in an oven at 180 °C for 12 h. After the reaction concluded, the autoclave was naturally cooled to room temperature. Subsequently, the electrode was washed with deionized water to remove residual ions, and finally dried at 60 °C for 8 h, yielding the WO_x_/FTO electrode.

**Figure 4:**
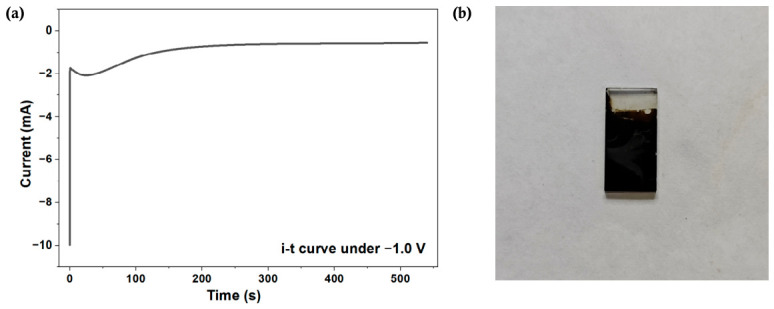
Potentiostatic electrodeposition i–t curve of the composite electrode at −1.0 V (**a**) and the fabricated Co_3_O_4_/WO_x_/FTO electrode (**b**).

A conventional three-electrode system (CHI660E, Shanghai Chenhua Instrument Co., Ltd., Shanghai, China). was employed to deposit cobalt oxide (Co_3_O_4_) onto the surface of the WO_x_/FTO electrode. A WO_x_/FTO electrode with dimensions of 1 cm × 2 cm was utilized as the working electrode, a 1.5 cm × 1.5 cm platinum plate as the counter electrode, and an Ag/AgCl electrode as the reference electrode. The electrolyte was prepared by dissolving 2.91 g of Co(NO_3_)_2_·6H_2_O in 60 mL of deionized water and 40 mL of ethylene glycol. Electrodeposition was performed by applying a constant voltage of −1.0 V at room temperature for a deposition time of 9 min. Figure 4a illustrates the potentiostatic electrodeposition current–time (i–t) curve during the fabrication of the composite electrode, reflecting the successful nucleation and stable growth of the electroactive material on the electrode surface. The resulting Co(OH)_2_/WO_x_/FTO electrode obtained after deposition was placed in a muffle furnace and annealed at 300 °C for 2 h with a heating rate of 3 °C/min, ultimately yielding the Co_3_O_4_/WO_x_/FTO electrode.

Figure 4b showcases a representative sample of the fabricated Co_3_O_4_/WO_x_/FTO electrodes. To ensure the reproducibility of the experiments and prevent potential surface degradation during continuous testing, a batch of identical electrodes was fabricated under the exact same conditions. Throughout the subsequent electrochemical measurements, multiple fresh electrodes from this batch were utilized. Since the core objective of the present work focuses on the circuit architecture of the photo-assisted electrochemical detection system, the composite electrodes were synthesized strictly for system functionality validation. For exhaustive physicochemical characterization of these electrodes, readers are referred to the previous work by Gao [37].

## 3. Experiments and Results

### 3.1. Performance Evaluation of the Analog Front-End

**Figure 5:**
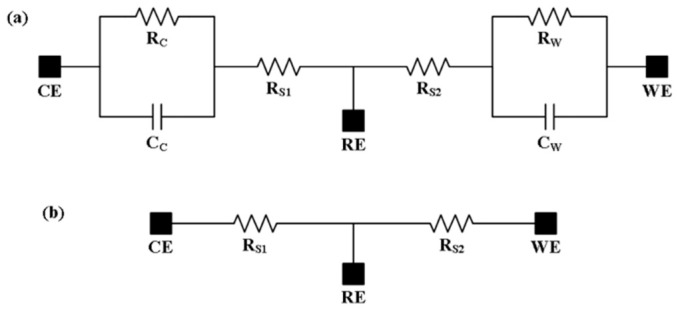
Equivalent impedance model (**a**) and simplified model (**b**) of the electrochemical cell, where the black squares represent the connection terminals for the electrodes.

The solution impedance of the electrochemical cell can be equivalent to the model shown in Figure 5a [30], where R_s1_ is the solution resistance between the CE and RE, and R_s2_ is the solution resistance between the WE and RE; R_C_ and R_W_ are the charge transfer resistances at the surfaces of the CE and WE, respectively; C_C_ and C_W_ are the electrical double-layer capacitances at the solution interface of the CE and WE, respectively. For DC signals, the equivalent model can be further simplified to Figure 5b. Based on this simplified model, a systematic performance evaluation of the designed electrochemical potentiostat AFE was conducted.

First, according to the DPV voltage range used for detecting copper ions, the DAC is selected to apply a constant potential of 0.6 V, at which point the potential difference between the WE and RE is synchronously held constant at 0.6 V. Different resistors are sequentially utilized to simulate the electrochemical cell. With the theoretical current value *I_ref_* as the horizontal axis and the actual measured current value *I_test_* as the vertical axis, a curve is fitted to evaluate the relative standard deviation. The conversion formula is as follows:

(4) $$I_{ref}=\frac{V_{WR}}{R_{dummy}}$$

(5) $$I_{test}=\frac{V_{ADC}}{R_{TIA}}$$

**Figure 6:**
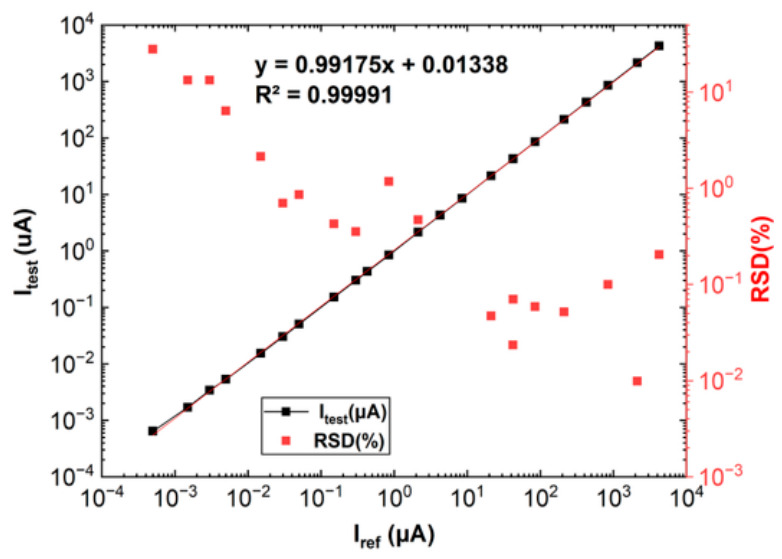
Measured current versus theoretical current and its relative standard deviation.

Here, *R_dummy_* is the resistor used to simulate the electrochemical cell, and *V_WR_* = *V_DAC_*. Figure 6 illustrates the measurement range and accuracy of the current detection system across the four gain ranges. It can be seen that the fitted curve is *I_test_* (μA) = 0.99175 *I_ref_* (μA) + 0.01338 (R^2^ = 0.99991), indicating that the designed potentiostat AFE exhibits excellent linearity and measurement accuracy over a wide dynamic range from 10 nA to 4 mA, with a weak current detection lower limit reaching 5 nA. This demonstrates that the instrument possesses the capability to accurately capture and convert microampere- and nanoampere-level weak Faradaic currents, fully satisfying the requirements for high-precision signal acquisition in trace electrochemical analysis.

Subsequently, a 4.7 kΩ resistor was utilized to simulate the electrochemical cell, and a simulated DPV scan was performed using the designed AFE. The DPV parameters were set as follows: initial potential −0.6 V, final potential 0.5 V, potential step 0.005 V, pulse amplitude 0.025 V, pulse period 0.20 s, and pulse width 0.10 s. Theoretically, the acquired differential current value *I_Diff_* should be:

(6) $$I_{Diff}=\frac{E_{H}}{R_{s}}-\frac{E_{L}}{R_{s}}=(E_{H}-E_{L})/R_{s}$$

**Figure 7:**
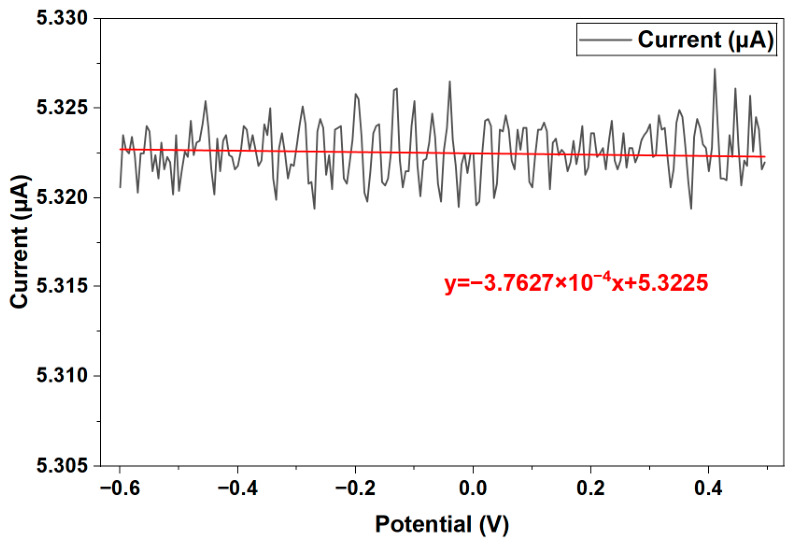
DPV test curve (black line) and linear fitting curve (red line) of the AFE for a 4.7 kΩ simulated electrochemical cell.

Herein, *E_L_* is the base potential when the DPV pulse is at a low level, *E_H_* is the pulse potential when the DPV pulse is at a high level, and *R_s_* is the simulated solution resistance. The value of (*E_H_* − *E_L_*) represents the pulse amplitude. Given that *R_s_* = 4.7 kΩ, theoretically, *I_Diff_* = 0.025 V/4.7 kΩ = 5.319 μA. Figure 7 presents the current curve acquired by the AFE. It can be observed that the average value of the measured current is 5.3225 μA. The absolute deviation from the theoretical value of 5.3191 μA is +0.0034 μA, corresponding to a relative deviation of 0.063%. The aforementioned tests indicate that the designed AFE possesses exceptionally high current measurement accuracy and outstanding DPV signal detection capabilities, fully satisfying the requirements for the electrochemical analysis of heavy metals.

### 3.2. Performance Evaluation of the LED Driver Circuit

#### 3.2.1. Output Power and Illumination Intensity Range

**Figure 8:**
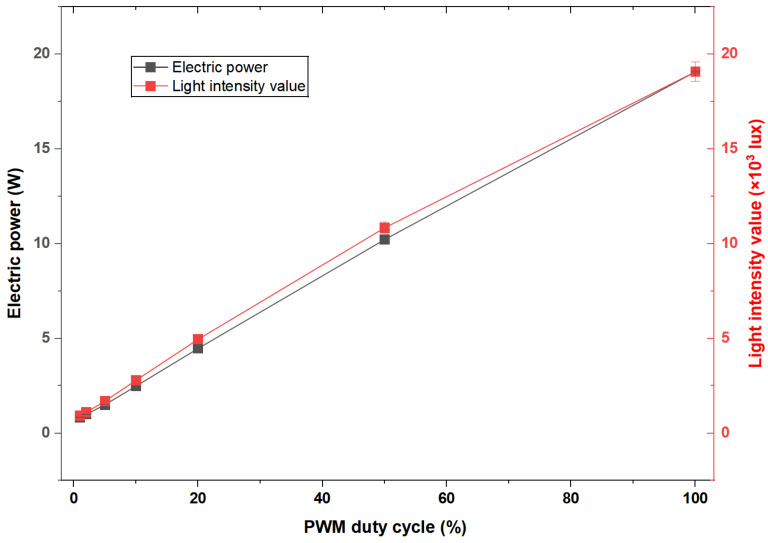
Input PWM duty cycle versus output electrical power and illumination intensity.

The LED light source was vertically positioned within a dark box. PWM waveforms with varying duty cycles were input into the LED driver circuit. The power was calculated based on the output voltage and current of the regulated power supply, and its relationship with the illumination intensity was evaluated. The light intensity sensor was placed at a distance of 10 cm from the light source, which corresponds to the position of the WE during the photo-assisted electrochemical testing process. An ammeter was connected in series within the LED driver circuit. The operating voltage of the LED is 12 V, and the power was calculated by measuring the current value. To prevent the high-power LED from damaging the operator’s eyesight, Wi-Fi wireless communication technology was employed. The duty cycle of the PWM waveform input to the LED driver circuit can be wirelessly adjusted on the PC terminal, thereby achieving regulation of the LED brightness. Figure 8 illustrates the corresponding relationship among the input PWM duty cycle, the output electrical power of the LED driver circuit, and the measured light intensity value, demonstrating that the system is capable of providing light intensity regulation over a wide dynamic range.

#### 3.2.2. Dimming PID Algorithm

To achieve more precise illuminance control at specific locations, the system introduces an incremental PID dimming algorithm [38] into the feedback loop, which can automatically adjust to the set illumination intensity. By utilizing this algorithm, operators are not required to manually search for the appropriate PWM duty cycle, thereby enhancing the convenience of the system and avoiding potential damage to the operators’ eyesight caused by the high-power LED light source. To balance response speed and dimming stability, the PID parameters were determined as K_p_ = 0.001, K_i_ = 0.0005, and K_d_ = 0.001.

**Figure 9:**
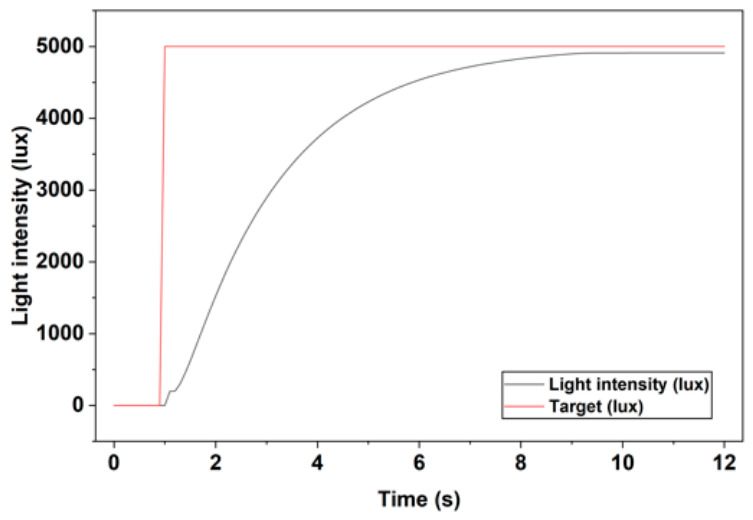
Step response of the incremental PID dimming algorithm.

Initially, a closed-loop simulation analysis of this algorithm was conducted on the Simulink platform. The discrete step response simulation results of the PID algorithm are illustrated in Figure 9. When a step target signal of 5000 lux is input at 1.0 s, the incremental limiting mechanism (ΔU_max_ = 5.0%) within the algorithm is triggered due to the large initial deviation, causing the control output to increase linearly at the maximum allowable step size. This results in a smooth linear climb of the light intensity between 1.0 s and 2.5 s, fundamentally eliminating the light source flickering phenomenon caused by excessive control current during the startup transient. As the system approaches the set value, the algorithm automatically transitions into an overdamped regulation state, ultimately gliding smoothly into the steady-state region with an excellent zero-overshoot characteristic. After 9.1 s, the system enters a designed deadband of ±100 lux, the historical errors are cleared, and the light intensity finally stabilizes precisely at 4909.16 lux.

In the actual dimming tests, the operator sends the target light intensity value from the PC terminal, and the MCU utilizes the PID algorithm to automatically calculate the increment and adjust the PWM duty cycle to drive the LED. Table 1 presents the actual light intensity values detected at the target location, as well as the time required for the illumination intensity to reach the set value from zero and the final PWM duty cycle. The results demonstrate that the actually achieved light intensity values all fell within the range of ±100 lux of the preset values, which complies with the ±100 lux deadband suppression of the PID algorithm. The entire dimming process was smooth with no obvious oscillation, and the dimming speed was relatively fast, providing a constant and reliable light field environment for the electrochemical detection.

### 3.3. Photo-Assisted Electrochemical Testing of Copper Ions

This paper employed a photo-assisted electrochemical strategy based on DPV for the quantitative analysis of copper ions. A standard three-electrode system was constructed for the testing, utilizing photoactive Co_3_O_4_/WO_x_/FTO as the WE, platinum foil as the CE, and a saturated calomel electrode (SCE) as the RE. An acetate buffer solution (ABS, 0.1 mol/L, pH = 5.0) was utilized as the supporting electrolyte to conduct actual tests on a series of copper ion concentrations. Instrument calibration and curve fitting were performed in comparison with CHI660E, followed by the detection of copper ions in actual solution samples.

#### 3.3.1. Validation of the Electrode’s Photo-Response to Blue Light

**Figure 10:**
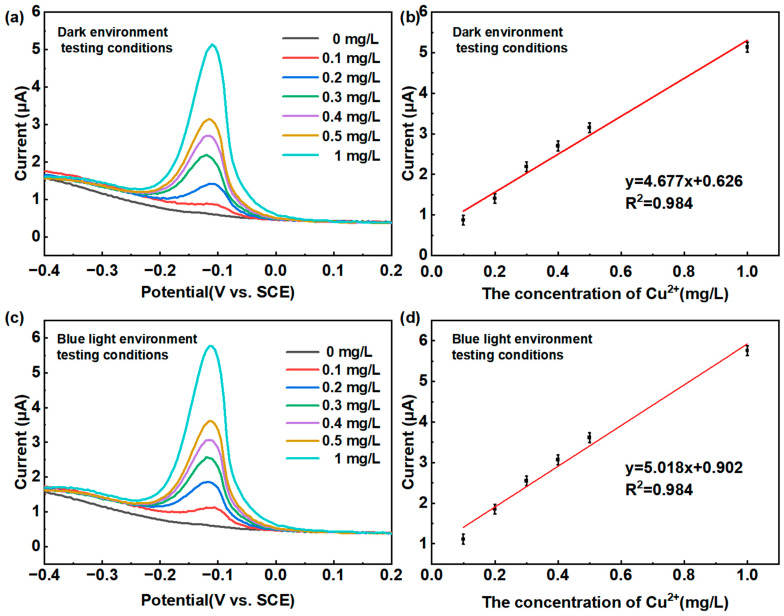
Validation of the electrode’s photo-response. (**a**) DPV responses and (**b**) calibration curve of the Co_3_O_4_/WO_x_/FTO electrode to different concentrations of copper ions under dark conditions; (**c**) DPV responses and (**d**) calibration curve under blue light illumination.

To demonstrate the enhancement mechanism of photoexcitation on sensing performance, a CHI660E electrochemical workstation was utilized to record the DPV responses and calibration curves of the WE in a 0.1–1 mg/L copper ion solution under dark conditions and 20 W blue LED illumination, respectively, as illustrated in Figure 10. By performing linear fitting and comparison on the two sets of voltammetric data, the linear regression equations between the Cu^2+^ concentration and the peak current (I_pa_) in the buffer solution under dark and blue light illumination conditions were I_pa_ (μA cm^−2^) = 46.75C (mg/L) + 1.61 (R^2^ = 0.981) and I_pa_ (μA cm^−2^) = 50.28C (mg/L) + 8.96 (R^2^ = 0.984), respectively. The results indicate that the stripping peak current of Cu^2+^ under blue light illumination exhibits an overall improvement compared to that under dark conditions. This proves that the fabricated electrode possesses a photo-response and further demonstrates that the relative performance enhancement of the photo-assisted electrochemical method is more significant at low concentrations.

#### 3.3.2. System Calibration with the CHI660

**Figure 11:**
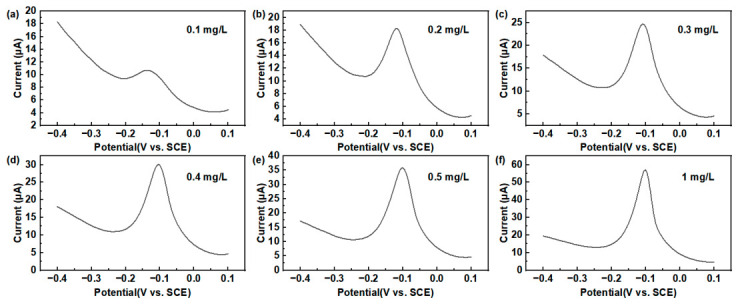
DPV response curves measured by the PAEcStat under different Cu^2+^ concentrations: (**a**) 0.1 mg/L, (**b**) 0.2 mg/L, (**c**) 0.3 mg/L, (**d**) 0.4 mg/L, (**e**) 0.5 mg/L, and (**f**) 1 mg/L.

After validating the photo-assisted mechanism, the PAEcStat was utilized to perform DPV voltammetric scanning on copper ion solutions with a concentration gradient ranging from 0.1 mg/L to 1 mg/L under identical blue light illumination conditions. The detailed parameter settings for DPV were as follows: initial potential −0.4 V, final potential 0.2 V, potential step 0.005 V, pulse amplitude 0.025 V, pulse period 0.50 s, and pulse width 0.25 s. Figure 11 displays the response curves obtained by the PAEcStat, and Table 2 presents the stripping peak currents at different Cu^2+^ concentrations in comparison with the CHI660E. The results demonstrate that the PAEcStat possesses a reliable level of detection accuracy.

#### 3.3.3. System Repeatability

**Figure 12:**
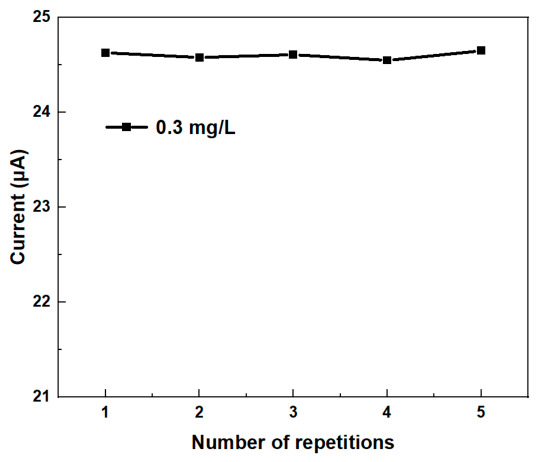
Repeatability of the proposed system with a solution containing 0.3 mg/L of Cu^2+^.

To evaluate the precision and reliability of the designed PAEcStat system, a repeatability test was systematically conducted. Five successive differential pulse voltammetry (DPV) measurements were performed on a 0.3 mg/L Cu^2+^ solution using the Co_3_O_4_/WO_x_/FTO electrode under identical blue-light illumination conditions. As depicted in Figure 12, the stripping peak currents exhibited excellent consistency across the five independent repetitions, with the current values remaining highly stable at approximately 24.6 μA. The relative standard deviation (RSD) of the measured peak currents was calculated to be 0.20%. This remarkably low RSD value confirms that the integrated PAEcStat platform avoids significant signal degradation during multiple continuous scans, demonstrating outstanding operational stability and high reproducibility for the reliable on-site detection of heavy metal ions.

#### 3.3.4. Real Water Sample Analysis

The practical application of the sensor is an important indicator for evaluating the system. Therefore, real water sample analysis was performed using local drinking water to evaluate the reliability of the PAEcStat. Briefly, the drinking water sample was mixed with 0.1 M acetate buffer solution (ABS, pH = 5.0) in a 1:1 volume ratio. The standard addition method was utilized to determine the concentration of Cu^2+^ in the water samples at 0.1 mg/L and 0.2 mg/L via DPV detection under blue-light illumination. As shown in Table 3, the results demonstrate that the recoveries ranged from 105.0% to 105.4%, with a relative standard deviation (RSD) lower than 6.31%. These results indicate that the proposed PAEcStat system combined with the Co_3_O_4_/WO_x_/FTO electrode exhibits high accuracy and can be effectively applied for the reliable on-site detection of Cu^2+^ in real environmental water samples.

## 4. Conclusions

In this paper, an integrated photo-assisted electrochemical potentiostat (PAEcStat) was designed and implemented for the rapid, on-site detection of heavy metal ions in water bodies. Regarding the hardware architecture, the system successfully integrates an electrochemical analog front-end (AFE) featuring high resolution and low noise, alongside a high-power LED driver circuit based on incremental PID closed-loop control. Hardware performance evaluations indicate that the system can not only achieve high-precision weak current measurements over a wide dynamic range from 10 nA to 4 mA, but also provide a stable excitation light field via negative feedback regulation. For detection application validation, a photoresponsive Co_3_O_4_/WO_x_/FTO sensing electrode was fabricated. Under blue light excitation, it bypassed the time-consuming preconcentration step inherent in traditional stripping voltammetry, thereby achieving the rapid and direct quantitative analysis of copper ions. Parallel comparative experiments conducted with a commercial benchtop electrochemical workstation confirmed that the voltammetric response curves obtained by the PAEcStat exhibited a good degree of consistency with those of the commercial instrument.

The PAEcStat system developed in this work successfully achieved the high precision and high integration of hardware required for photo-assisted electrochemical analysis. Through systematic simulated electrochemical cell experiments and rigorous hardware evaluations, it was fully verified that the system possesses exceptionally high accuracy and reliability in weak current detection. Simultaneously, combined with actual photoelectrochemical detection experiments, its effectiveness in sensing applications was further validated. This work effectively overcomes the limitations of traditional photo-assisted testing, which heavily relies on bulky benchtop equipment, and provides a robust engineering hardware reference scheme for transitioning photo-assisted electrochemical sensing technology from theoretical laboratory research to practical outdoor applications.

## Figures and Tables

**Table 1:** Actual light intensity values at the target location, dimming time, and reached PWM duty cycles.

Set Light Intensity (lux)	Actual Light Intensity (lux)	Dimming Time (s)	PWM Duty Cycle (%)
1000	1075	3.05	1
2000	1934	5.26	4
5000	4925	10.67	17
9000	8936	12.13	34
14,000	13,914	14.71	58
19,000	18,910	17.18	85

**Table 2:** Peak currents detected by the CHI660E and PAEcStat under different Cu^2+^ concentrations.

Cu^2+^ Concentration (mg/L)	CHI660E (μA)	PAEcStat (μA)
0.1	11.12	10.71
0.2	18.54	18.28
0.3	25.47	24.67
0.4	30.68	30.12
0.5	36.15	35.89
1.0	57.55	57.12

**Table 3:** Recovery tests of Cu^2+^ in drinking water samples using the PAEcStat system.

Sample	Spiked (mg/L)	Found (mg/L)	Recovery (%)	RSD (%, *n* = 3)
Drinking water	0	Nd *	-	-
	0.1	0.105	105	4.62
	0.2	0.211	105.4	6.31

* Nd: Not detected.

## Data Availability

The data presented in this study are not publicly available due to confidentiality and privacy restrictions.
